# Should the two‐trial paradigm still be the gold standard in drug assessment?

**DOI:** 10.1002/pst.2262

**Published:** 2022-08-26

**Authors:** Stella Jinran Zhan, Cornelia Ursula Kunz, Nigel Stallard

**Affiliations:** ^1^ Warwick Medical School University of Warwick Coventry UK; ^2^ Biostatistics and Data Sciences Boehringer Ingelheim Pharma GmbH & Co. KG Biberach/Riss Germany

**Keywords:** pivotal trials, pooled analysis, power, regulatory guideline, type I error control

## Abstract

Two significant pivotal trials are usually required for a new drug approval by a regulatory agency. This standard requirement is known as the two‐trial paradigm. However, several authors have questioned why we need exactly two pivotal trials, what statistical error the regulators are trying to protect against, and potential alternative approaches. Therefore, it is important to investigate these questions to better understand the regulatory decision‐making in the assessment of drugs' effectiveness. It is common that two identically designed trials are run solely to adhere to the two‐trial rule. Previous work showed that combining the data from the two trials into a single trial (one‐trial paradigm) would increase the power while ensuring the same level of type I error protection as the two‐trial paradigm. However, this is true only under a specific scenario and there is little investigation on the type I error protection over the whole null region. In this article, we compare the two paradigms by considering scenarios in which the two trials are conducted in identical or different populations as well as with equal or unequal size. With identical populations, the results show that a single trial provides better type I error protection and higher power. Conversely, with different populations, although the one‐trial rule is more powerful in some cases, it does not always protect against the type I error. Hence, there is the need for appropriate flexibility around the two‐trial paradigm and the appropriate approach should be chosen based on the questions we are interested in.

## 1 INTRODUCTION

The standard effectiveness requirement for a new drug approval, explicitly presented in the 1998 FDA guidance, is often the conduct of “at least two pivotal, adequate, and well‐controlled trials,” each with a two‐sided alpha level of 0.05. This two‐trial paradigm or rule is presented to fulfill “the need for independent substantiation of experimental results.”^1^

However, an increasing number of submissions are presenting alternatives to this traditional two‐trial rule, for example, those involving COVID‐19 vaccines and Aducanumab. For their vaccine, Astrazeneca/Oxford have presented results from a pooled analysis of the combined data of phase II/III and phase III trials,^2^ whereas both Pfizer/BioNTech^3^ and Moderna^4^ conducted a single pivotal trial. The recent approval of the first Alzheimer's drug, Aducanumab, has also raised multiple questions linked to this regulatory requirement. Two identical phase III trials were designed and conducted to prove the efficacy of the drug, but their results turned out to be one positive and one negative. In spite of this inconclusive result, the FDA granted a temporary license for the drug through the “accelerated approval” program. Although this example is more complicated than what is described here, for example, trial termination for futility and post hoc analysis, this decision has led to lots of controversies and shows that the regulatory authorities are more flexible than they might have been, hence challenging the standard FDA two‐trial paradigm.^5^

These examples confirm the need to reconsider the two‐trial paradigm and understand what the regulators are looking for in the assessment of drugs' effectiveness. Sometimes, it is common that two separate trials are conducted for no other reason than adhering to this two‐trial standard. In this case, the trial protocols can be almost identical and discussions have been on whether a combined analysis of the data from the two individual trials would be appropriate to demonstrate drug's effectiveness from a statistical point of view. Conducting two identically designed trials can be considered one way of replicating the experiment, but these results might be influenced by the specific design and bring some systematic biases. If the aim is to obtain more informative and generalizable conclusions, the two trials could be run using different study populations (e.g., mild or severe patients), or distinct but related clinical endpoints or separate clinical sites.

Although this two‐trial rule has remained the conventional approach since the publication of the 1998 FDA guidance, more and more challenges are rising in rare diseases or life‐threatening diseases trials, where a second trial would be unfeasible or unethical. In addition, more innovative trial designs are emerging in the literature, therefore there is a need for appropriate flexibility around the standard two‐trial paradigm. It has become more frequent that a conditional approval of a new drug is issued after a positive trial result, which is followed by a second post‐marketing trial to continue the evaluation of its safety and efficacy.^6^ Both the FDA^1^, ^7^ and EMA^8^ have outlined some exemptions to the two‐trial rule, where one single pivotal trial is sufficient, for example, using a large multi‐center trial or one adequate and well‐controlled trial with confirmatory evidence to establish effectiveness. These types of exemptions highlight a continuous change in the characteristics and number of pivotal trials supporting drug approval over the past decades.^6^, ^9^ There is interest in how comparable it is to obtain the data from a single large pivotal trial instead of two. In addition, based on the Aducanumab example, people might be tempted to select one significant trial out of two or more trials that have been conducted, as the one on which the approval should be based. These are all different procedures and consequentially their properties will not be the same. In this article, we will focus on the comparison between the standard two‐trial paradigm and the one‐trial paradigm, modeled by combining data from two trials (more details are given in Section 2).

### 1.1 Statistical considerations

In the case of a single trial, the regulatory agencies would require more stringent criteria compared to the two‐trial rule to provide a “very persuasive finding” that might be interpreted as a “very low *p*‐value,” but no standard has yet been set for that.^10^ Various researchers have addressed the question on the difference between having one single trial and two independent trials, both as a regulatory strategy and also from a statistical point of view.

Previous work by Fisher^11^ suggested that a single study at the two‐sided significance level of 0.00125 (that is equivalent to a one‐sided level of 0.025^2^) does provide the same overall type I error as the two‐trial rule. Later work by Maca et al.^12^ and Shun et al.^13^ has addressed the statistical aspects in the standard two‐trial paradigm compared with the one‐trial paradigm, including statistical assumptions, type I error, and power. They pointed out two main statistical assumptions regarding the homogeneity and heterogeneity of the populations in the trials, which would affect the difference between conducting one or two pivotal trials. Their research suggested that the one‐trial paradigm is applicable only in the presence of a homogeneous population and more convincing results are required to provide the same level of evidence as the two‐trial paradigm. Methods for integrating data and pooling *p*‐values (meta‐analysis) are also presented. Modified versions of the two‐trial rule have been proposed in Maca et al.^12^ and Rosenkranz.^14^

Existing literature has been mainly focused on the discussion of power gain with the one‐trial paradigm while ensuring the same level of type I error protection as the two‐trial rule at a single point of the null region. Some authors^12^, ^13^ have also included discussion on sample size calculation when comparing the different approaches, therefore this is not covered here. However, there is little investigation on the type I error protection over the whole null region (more details are given in Section 2.2) and the influence of the population heterogeneity on the operating characteristics.

In this article, we give a clearer picture of the comparison between the two paradigms. We consider scenarios where the two trials are conducted in identical or different populations as well as when they have equal or unequal size. Section 2 provides the general setting and notation with details of the hypotheses tested and test statistics. It is followed by the comparison based on rejection regions and error rates for different scenarios in Section 3. In particular, we show that under the scenario of identical populations, the one‐trial approach has lower type I error rate and higher power compared to the two‐trial approach. Whereas, under the scenario of different populations, the one‐trial rule is more powerful in some cases, but it does not always protect against the type I error. Finally, we conclude with the discussion in Section 4.

## 2 GENERAL SETTING

The two‐trial paradigm aims to avoid unanticipated, systematic biases, reduce type I error rate, and dependency on site‐specific factors.^1^ The conduct of the two trials in different study populations or separate clinical sites would be preferred when we are interested in knowing the conclusions in an overall (more general) population. In these cases, we would expect that the drug might have different effects in the two trials. For this reason, in our comparison we consider two scenarios:

1. The treatment effects in the two trial populations are identical.
2. The treatment effects in the two trial populations are different.

Here, we will use the term “identical populations” to refer to scenario (1) with identical treatment effects in the two populations.

The focus of this article is the comparison between two approaches under the two scenarios listed above: the standard two‐trial paradigm and the one‐trial paradigm, modeled by combining data from two trials which may be from identical or different populations. Particular attention should be paid to the one‐trial paradigm in the presence of two populations, which could be interpreted in two ways: two independent trials are already conducted in two populations and then we combine them, or we run one single trial with patients randomly drawn from two populations. In this article, we discuss the former case, where the numbers of patients from the two populations are fixed by the trial sample sizes. While, in the latter case, we would have random numbers of patients.

### 2.1 Notation

We are interested in comparing two treatments (experimental vs. control) in Phase III clinical trials. Under the standard regulatory requirement, we need to conduct two pivotal trials and both have to be significant at the one‐sided *α*∕2 = 0.025 level. Assume that we have *N* patients in total, which are split into two trials with *N*_1_ = *fN* in the first Phase III trial and *N*_2_ = (1 − *f*)*N* in the second, where 0 < *f* < 1 is a value fixed in advance, perhaps chosen to reflect some aspects that we believe about the populations (e.g., their prevalence). Patients are randomized in a 1:1 ratio between the two arms, so that *n*_1_ = *fN*∕2 and *n*_2_ = (1 − *f*)*N*∕2 patients are enrolled in each arm of the first and second trial, respectively.

Let *X*_*ij*_ denote the data from patient *j* from both populations, with patients *j* = 1, …, *n*_1_ in population 1 and patients *j* = *n*_1_ + 1, …, *n*_1_ + *n*_2_ in population 2, in treatment *i* for *i* = 0 (control) and *i* = 1 (experimental). Suppose that they are independent and normally distributed as

$$\begin{array}{l} X_{\mathrm{ij}}∼N\left(\mu_{i1},\sigma^{2}\right)\;j=1,\ldots ,n_{1},\\ X_{\mathrm{ij}}∼N\left(\mu_{i2},\sigma^{2}\right)\;j=n_{1}+1,\ldots ,n_{1}+n_{2}, \end{array}$$

where *μ*_*i*1_ and *μ*_*i*2_ indicate the true means of the two trial populations for treatment *i* and *σ* ^2^ denote the true variance, which is assumed to be known and equal to 1 in the two populations. Let Δ_1_ = *μ*_11_ − *μ*_01_ and Δ_2_ = *μ*_12_ − *μ*_02_ the treatment difference for the first and second trial populations. Since we are assuming that the variance is equal to 1, the two Δ_*i*_ are equivalent to the corresponding Cohen's *d* values.

Under scenario (1), we can assume equal treatment means for the two trial populations: *μ*_11_ = *μ*_12_ and *μ*_01_ = *μ*_02_, which leads to Δ_1_ = Δ_2_. While, under scenario (2), we would have different treatment differences Δ_1_ ≠ Δ_2_.

In the one‐trial paradigm, we are interested in testing a single parameter, the average treatment effect $\tilde{\Delta }$ = *f*(*μ*_11_ − *μ*_01_) + (1 − *f*)(*μ*_12_ − *μ*_02_) = *f*Δ_1_ + (1 − *f*)Δ_2_, with *f* as above. Under scenario (1), we can assume that the data are normally distributed with common population means *μ*_11_ = *μ*_12_ and *μ*_01_ = *μ*_02_:

$$X_{\mathrm{ij}}∼N\left(\mu_{i1},\sigma^{2}\right)\;j=1,\ldots ,n_{1},n_{1}+1,\ldots ,n_{1}+n_{2}$$

and $\tilde{\Delta }$= Δ_1_ = *μ*_11_ − *μ*_01_, note that $\tilde{\Delta }$ is not influenced by *f* when we assume that the data of the two trials are from identical populations. Based on the two scenarios listed above, we can investigate the statistical properties of the two‐trial and one‐trial paradigms in the presence of identical or different populations. First, in the next section, we provide details of the hypotheses tested and test statistics in the two paradigms.

An alternative way to express the difference between the treatment effects in different trials is to use a meta‐analysis framework in which we consider a common effect Δ and introduce a parameter *τ* ^2^ to measure the degree of heterogeneity between the trials. Under scenario (1), we assume that there is no difference between the individual trials, therefore the effect for each individual trial Δ_*k*_ with *k* = 1,2 is identical to the common effect Δ and *τ* ^2^ is equal to 0, while under scenario (2) we assume that there is heterogeneity and *τ* ^2^ > 0. Under the random‐effects model, we can assume that

$$\begin{array}{l} {\hat{\Delta }}_{k}∼N\left(\Delta_{k},s_{k}^{2}\right)\\ \Delta_{k}∼N\left(\Delta ,\tau^{2}\right) \end{array}$$

where the variance $s_{k}^{2}$ is treated as known and *τ* ^2^ can be estimated and treated as fixed. To obtain the estimate of the true common effect Δ, we use a weighted average of all the trials. The weights are determined by taking the inverse of the variance of the effect for each study and adjusting to incorporate the variance of the distribution of true effects *τ* ^2^: $w_{k}=1/\left(s_{k}^{2}+\tau^{2}\right)$. The estimate of the common effect becomes

$$\hat{\Delta }=\frac{\sum_{k=1}^{K}{\hat{\Delta }}_{k}w_{k}}{\sum_{k=1}^{K}w_{k}}.$$

where ${\hat{\Delta }}_{k}$ are the estimates for the individual parameters Δ_*k*_ of each trial and *K* = 2 is the total number of trials.

### 2.2 Hypotheses tested

The difference between the two approaches under the two scenarios becomes clear when we consider the hypotheses tested. Starting with the regulatory requirement of the two‐trial rule, we perform two tests, one for each trial. The individual hypotheses tested in the two trials are $H_{0}^{\left(1\right)}:\Delta_{1}\leq 0$ and $H_{0}^{\left(2\right)}:\Delta_{2}\leq 0$. To obtain approval, we need both trials to be significant, this means that we need to reject both individual hypotheses $H_{0}^{\left(1\right)}$ and $H_{0}^{\left(2\right)}$. Therefore, our objective is to test the overall hypothesis $H_{0}=H_{0}^{\left(1\right)}\cup H_{0}^{\left(2\right)}$ versus $H_{a}=H_{a}^{\left(1\right)}\cap H_{a}^{\left(2\right)}$, where the overall null hypothesis is given by the union of the two individual null hypotheses and the overall alternative hypothesis is the intersection of the individual alternative hypotheses. Under scenario (1), since the two individual trials are conducted in identical populations (Δ_1_ = Δ_2_), the same individual hypothesis is tested twice ($H_{0}^{\left(1\right)}=H_{0}^{\left(2\right)},\;H_{a}^{\left(1\right)}=H_{a}^{\left(2\right)}$) so that the overall hypotheses become

$$H_{0}=H_{0}^{\left(1\right)}\cup H_{0}^{\left(2\right)}=H_{0}^{\left(1\right)}\cap H_{0}^{\left(2\right)}=H_{0}^{\left(1\right)}\;H_{a}=H_{a}^{\left(1\right)}\cap H_{a}^{\left(2\right)}=H_{a}^{\left(1\right)}.$$

Note that in this case, the union of the individual null hypotheses is equal to their intersection, which will not be the case under scenario (2).

In the one‐trial paradigm, we test a single hypothesis whether we believe that scenario (1) is true or not. Our interest will not be in testing the individual hypotheses rather the overall hypothesis:

$$H_{0}:\tilde{\Delta }\leq 0\;H_{a}:\tilde{\Delta }>0.$$

Under scenario (1), $\tilde{\Delta }$ reduces to Δ_1_ = *μ*_11_ − *μ*_01_ and the overall hypothesis *H*_0_ corresponds to the same overall hypothesis in the two‐trial paradigm with identical populations $H_{0}^{\left(1\right)}$.

**FIGURE 1:**
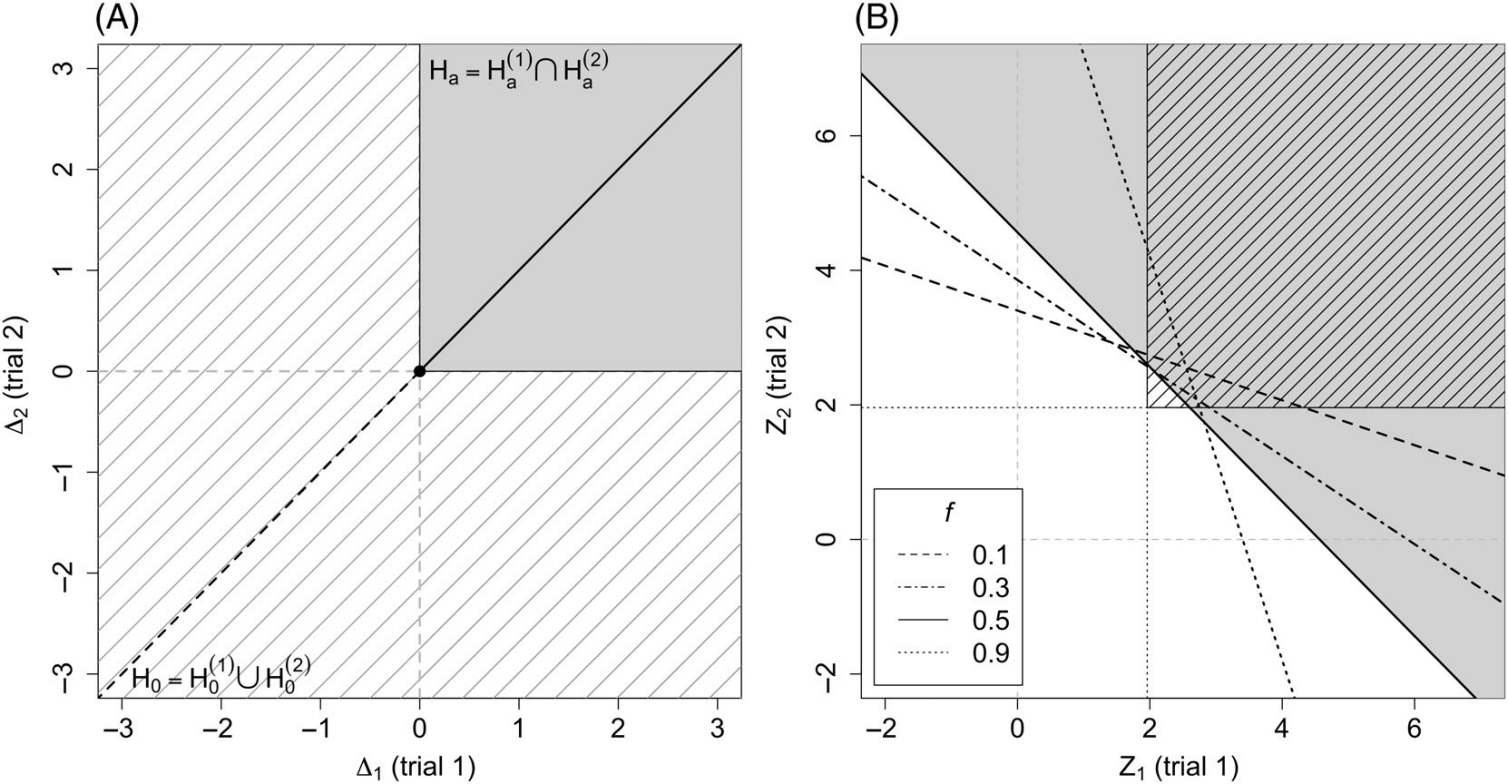
(A) Null hypothesis and alternative hypothesis regions (B) rejection regions for the two‐trial paradigm (striped area) and one‐trial paradigm (grey area) for *f* = 0.5. Oblique lines with different line types indicate the boundaries for the one‐trial paradigm with different *f* values

The null and alternative hypothesis regions for the two approaches are shown in Figure 1a. Under scenario (1), the two regions reduce to the diagonal line, with the alternative region indicated by the solid black line and the null region by the dashed black line. Note that this is the same for both the two‐trial and one‐trial paradigms. Under scenario (2) of different populations, for the two‐trial paradigm, we can identify the alternative hypothesis region as the upper‐right quadrant in grey and the null hypothesis region is made up of the remaining three quadrants with striped grey lines.

The hypotheses and parameter space for the two‐trial paradigm and one‐trial paradigm are summarized in Tables 1 and 2, separated in cases where we have identical or different populations.

**TABLE 1:** Statistical considerations in the two‐trial paradigm

	Two‐trial paradigm	
	**Identical populations**	**Different populations**
**Individual hypotheses**	1. $H_{0}^{\left(1\right)}:\Delta_{1}\leq 0$ $H_{a}^{\left(1\right)}:\Delta_{1}>0$	1. $H_{0}^{\left(1\right)}:\Delta_{1}\leq 0$ $H_{a}^{\left(1\right)}:\Delta_{1}>0$
	2. $H_{0}^{\left(2\right)}:\Delta_{1}\leq 0$ $H_{a}^{\left(2\right)}:\Delta_{1}>0$	2. $H_{0}^{\left(2\right)}:\Delta_{2}\leq 0$ $H_{a}^{\left(2\right)}:\Delta_{2}>0$
**Overall hypothesis**	REQUIRE: Both significant trials $H_{0}=H_{0}^{\left(1\right)}\cup H_{0}^{\left(1\right)}=H_{0}^{\left(1\right)}\cap H_{0}^{\left(1\right)}=H_{0}^{\left(1\right)}$ $H_{a}=H_{a}^{\left(1\right)}\cap H_{a}^{\left(1\right)}=H_{a}^{\left(1\right)}$ *Note*: $H_{0}^{\left(1\right)}=H_{0}^{\left(2\right)}$ and $H_{a}^{\left(1\right)}=H_{a}^{\left(2\right)}$ (the same hypothesis is tested twice)	REQUIRE: Both significant trials $H_{0}=H_{0}^{\left(1\right)}\cup H_{0}^{\left(2\right)}$ $H_{a}=H_{a}^{\left(1\right)}\cap H_{a}^{\left(2\right)}$
**Parameter space**	Under $H_{0}:\;\left\{\Delta_{1}\;\in \left(-\infty ,\;0\right]\right\}$	Under $H_{0}:\;\left\{\Delta_{1}\;\in \left(-\infty ,\;0\right],\;\Delta_{2}\;\in \left(-\infty ,\infty \right)\right\}\;\cup \;\left\{\Delta_{1}\;\in \left(-\infty ,\infty \right),\;\Delta_{2}\;\in \left(-\infty ,\;0\right]\right\}$

**TABLE 2:** Statistical considerations in the one‐trial paradigm. [Correction added on 7 September 2022, after first online publication: The duplicate equations on Table 2 were removed in this version.]

	One‐trial paradigm	
	Identical populations	Different populations
**Individual hypotheses**	‐	‐
**Overall hypothesis**	$H_{0}:\;\tilde{\Delta }\leq 0\;\;H_{a}:\;\tilde{\Delta }>0$	$H_{0}:\;\tilde{\Delta }\leq 0\;\;H_{a}:\;\tilde{\Delta }>0$
**Parameter space**	Under $H_{0}:\;\left\{\tilde{\Delta }\;\in \left(-\infty ,\;0\right]\right\}$	Under $H_{0}:\;\left\{\tilde{\Delta }\;\in \left(-\infty ,\;0\right]\right\}$

Under the assumption of a random‐effects model, we test a single hypothesis on the common effect which is different from the study‐specific hypothesis tests. Hence, the null hypothesis is that the mean of the true effects Δ is less than zero:

$$H_{0}:\Delta \leq 0\;\mathrm{vs}H_{a}:\Delta >0$$

with Δ as defined in Section 2.1.

### 2.3 Test statistics

Let *Z*_1_, *Z*_2_, and *Z* denote the test statistics for the comparison between the control and treatment for the two independent trials and the single trial based on the combined data. Assuming that the variance is known, they are given by

$$\begin{array}{l} Z_{1}=\frac{\frac{1}{n_{1}}\left(\sum_{j=1}^{n_{1}}X_{1j}-\sum_{j=1}^{n_{1}}X_{0j}\right)}{\sqrt{\frac{2\sigma^{2}}{n_{1}}}},\\ Z_{2}=\frac{\frac{1}{n_{2}}\left(\sum_{j=n_{1}+1}^{n_{1}+n_{2}}X_{1j}-\sum_{j=n_{1}+1}^{n_{1}+n_{2}}X_{0j}\right)}{\sqrt{\frac{2\sigma^{2}}{n_{2}}}},\\ Z=\frac{\frac{1}{n_{1}+n_{2}}\left(\sum_{j=1}^{n_{1}+n_{2}}X_{1j}-\sum_{j=1}^{n_{1}+n_{2}}X_{0j}\right)}{\sqrt{\frac{2\sigma^{2}}{n_{1}+n_{2}}}}=\sqrt{f}Z_{1}+\sqrt{1-f}Z_{2}. \end{array}$$

In this case, *Z*, the test statistics of the one‐trial approach calculated on the combined data, is equivalent to that provided by the inverse normal combination test.^15^ *Z*_1_, *Z*_2_, and *Z* follow the multivariate normal distribution with:

$$\left(\begin{array}{c} Z_{1}\\ Z_{2}\\ Z \end{array}\right)∼N\left[\left(\begin{array}{c} \delta_{1}\\ \delta_{2}\\ \delta \end{array}\right),\;\left(\begin{array}{ccc} 1 & 0 & \sqrt{f}\\ 0 & 1 & \sqrt{1-f}\\ \sqrt{f} & \sqrt{1-f} & 1 \end{array}\right)\right],\;\text{where}\left(\begin{array}{c} \delta_{1}\\ \delta_{2}\\ \delta \end{array}\right)=\left(\begin{array}{c} \frac{\mu_{11}-\mu_{01}}{\sqrt{2\sigma^{2}}}\sqrt{\frac{\mathrm{fN}}{2}}\\ \frac{\mu_{12}-\mu_{02}}{\sqrt{2\sigma^{2}}}\sqrt{\frac{\left(1-f\right)N}{2}}\\ \frac{f\left(\mu_{11}-\mu_{01}\right)+\left(1-f\right)\left(\mu_{12}-\mu_{02}\right)}{\sqrt{2\sigma^{2}}}\sqrt{\frac{N}{2}} \end{array}\right)$$

We note that the non‐centrality parameters *δ*_1_, *δ*_2_, and *δ* are related to Δ_1_, Δ_2_, and $\tilde{\Delta }$ as follows

$$\left(\begin{array}{c} \delta_{1}\\ \delta_{2}\\ \delta \end{array}\right)=\left(\begin{array}{c} \frac{\Delta_{1}}{\sqrt{2\sigma^{2}}}\sqrt{\frac{\mathrm{fN}}{2}}\\ \frac{\Delta_{2}}{\sqrt{2\sigma^{2}}}\sqrt{\frac{\left(1-f\right)N}{2}}\\ \frac{\tilde{\Delta }}{\sqrt{2\sigma^{2}}}\sqrt{\frac{N}{2}} \end{array}\right),\;\text{where}\;\tilde{\Delta }=f\Delta_{1}+\left(1-f\right)\Delta_{2}$$

Under scenario (1) with Δ_1_ = Δ_2_, all the three parameters ${\left(\delta_{1},\;\delta_{2},\;\delta \right)}^{T}$ are equal to $\Delta_{1}/\sqrt{2\sigma^{2}}$ scaled by the respective sample sizes.

In the random‐effects model, the between‐trial variability is usually estimated and incorporated in the weights $w_{k}$ as seen in Section 2.1. The most common estimators of *τ* ^2^ are DerSimonian‐Laird (“DL”), Restricted Maximum Likelihood (“REML”), or Maximum Likelihood (“ML”). Most of the time the uncertainty in the estimation is ignored and can become an issue in particular when we have only two trials. Detailed discussion can be found in Veroniki et al.^16^ and Friede et al.^17^ The test statistics for the random‐effects model *Z*_*RE*_ can be calculated as

$$Z_{\mathrm{RE}}=\frac{\hat{\Delta }}{\sqrt{\frac{1}{\sum_{k=1}^{K}w_{k}}}}=\frac{\hat{\Delta }}{\sqrt{\frac{1}{\sum_{k=1}^{K}\frac{1}{s_{k}^{2}+\tau^{2}}}}}$$

## 3 RESULTS

After outlining the test statistics of the two paradigms, in this section we present the results of the comparison between the two‐trial and one‐trial paradigms in terms of rejection regions and error rates.

### 3.1 Rejection regions

We consider one‐sided tests at *α*∕2 = 0.025. Under the two‐trial rule, we require both test statistics *Z*_1_ and *Z*_2_ to be larger than Φ^−1^(1 − *α*∕2). Under the one‐trial rule, different authors^11^, ^12^, ^13^ suggested that we could conduct one trial at one‐sided size *α* ^*'*^∕2 = (*α*∕2)^2^ = 0.000625 to ensure equal overall type I error rate to the two‐trial case, when Δ_1_ = Δ_2_ = $\tilde{\Delta }$ = 0. Therefore, we would require $Z>\Phi^{-1}\left(1-{\left(\alpha /2\right)}^{2}\right)$. The rejection regions for the two‐trial (Ω_*two*_) and the one‐trial (Ω_*one*_) rules become

$$\begin{array}{l} \Omega_{\mathrm{two}}=\left\{\left(Z_{1},Z_{2}\right):\left\{Z_{1}>\Phi^{-1}\left(1-\alpha /2\right)\right\}\cap \left\{Z_{2}>\Phi^{-1}\left(1-\alpha /2\right)\right\}\right\},\\ \Omega_{\mathrm{one}}=\left\{Z=\sqrt{f}Z_{1}+\sqrt{1-f}Z_{2}:Z>\Phi^{-1}\left(1-{\left(\alpha /2\right)}^{2}\right)\right\}. \end{array}$$

It is clear that the two regions are different and the one for the one‐trial depends on *f*. Figure 1b shows the rejection region for the two‐trial rule (striped area) and the one‐trial rule (grey area) when the two independent trials have the same size (*f* = 0.5). The boundaries for the one‐trial rule with different values of *f* are also included and indicated by different line types. In particular, the single trial rejection region corresponds to the area above the black line given by $Z_{2}>\Phi^{-1}\left(1-{\left(\alpha /2\right)}^{2}\right)/\sqrt{0.5}-Z_{1}$. If a pair of (*Z*_1_, *Z*_2_) results to be in the white shaded triangle below the black line, the two‐trial rule would declare success but the single trial would not. On the other side, if (*Z*_1_, *Z*_2_) is within the grey non‐striped area, the one‐trial rule leads to the rejection of *H*_0_, but the two‐trial rule would not.

For the random‐effects model, to ensure that its type I error rate is equal to the two‐trial rule, we can define its rejection region as

$$\Omega_{\mathrm{RE}}=\left\{Z_{\mathrm{RE}}:Z_{\mathrm{RE}}>\Phi^{-1}\left(1-{\left(\alpha /2\right)}^{2}\right)\right\}$$

### 3.2 Type I error and power

In this section, we will compare the operating characteristics (type I error and power) of the one‐trial and two‐trial rules. Firstly, we consider the results under the scenario of identical populations and secondly, under the scenario of different populations, both with equal and unequal sizes. Using the distribution of the test statistics given in Section 2.3, the probability of rejecting the overall hypothesis *H*_0_ for the two‐trial (*p*_*two*_) and one‐trial (*p*_*one*_) rules, as detailed in Maca et al.,^12^ is given by

$$\begin{array}{l} p_{\mathrm{two}}=P\left(\text{reject}\;H_{0}\right)=P\left(\text{reject}\;H_{0}^{\left(1\right)}\;\text{AND}\;H_{0}^{\left(2\right)}\right)=P\left(\left(Z_{1},\;Z_{2}\right)\in \Omega_{\mathrm{two}}\right)\\ \;=P\left\{Z_{1}>\Phi^{-1}\left(1-\alpha /2\right)\right\}P\left\{Z_{2}>\Phi^{-1}\left(1-\alpha /2\right)\right\}\\ \;=\left[1-\Phi \left\{\Phi^{-1}\left(1-\alpha /2\right)-\delta_{1}\right\}\right]\left[1-\Phi \left\{\Phi^{-1}\left(1-\alpha /2\right)-\delta_{2}\right\}\right],\\ p_{\mathrm{one}}=P\left(\text{reject}\;H_{0}\right)=P\left(Z\in \Omega_{\mathrm{one}}\right)\\ \;=P\left[Z>\Phi^{-1}\left\{1-{\left(\alpha /2\right)}^{2}\right\}\right]\\ \;=1-\Phi \left[\Phi^{-1}\left\{1-{\left(\alpha /2\right)}^{2}\right\}-\delta \right]. \end{array}$$

In Section 3.2.3, we will present simulation results for the type I error and power based on the random‐effects model for different values of the heterogeneity parameter *τ* ^2^.

#### 3.2.1 Two identical populations

**FIGURE 2:**
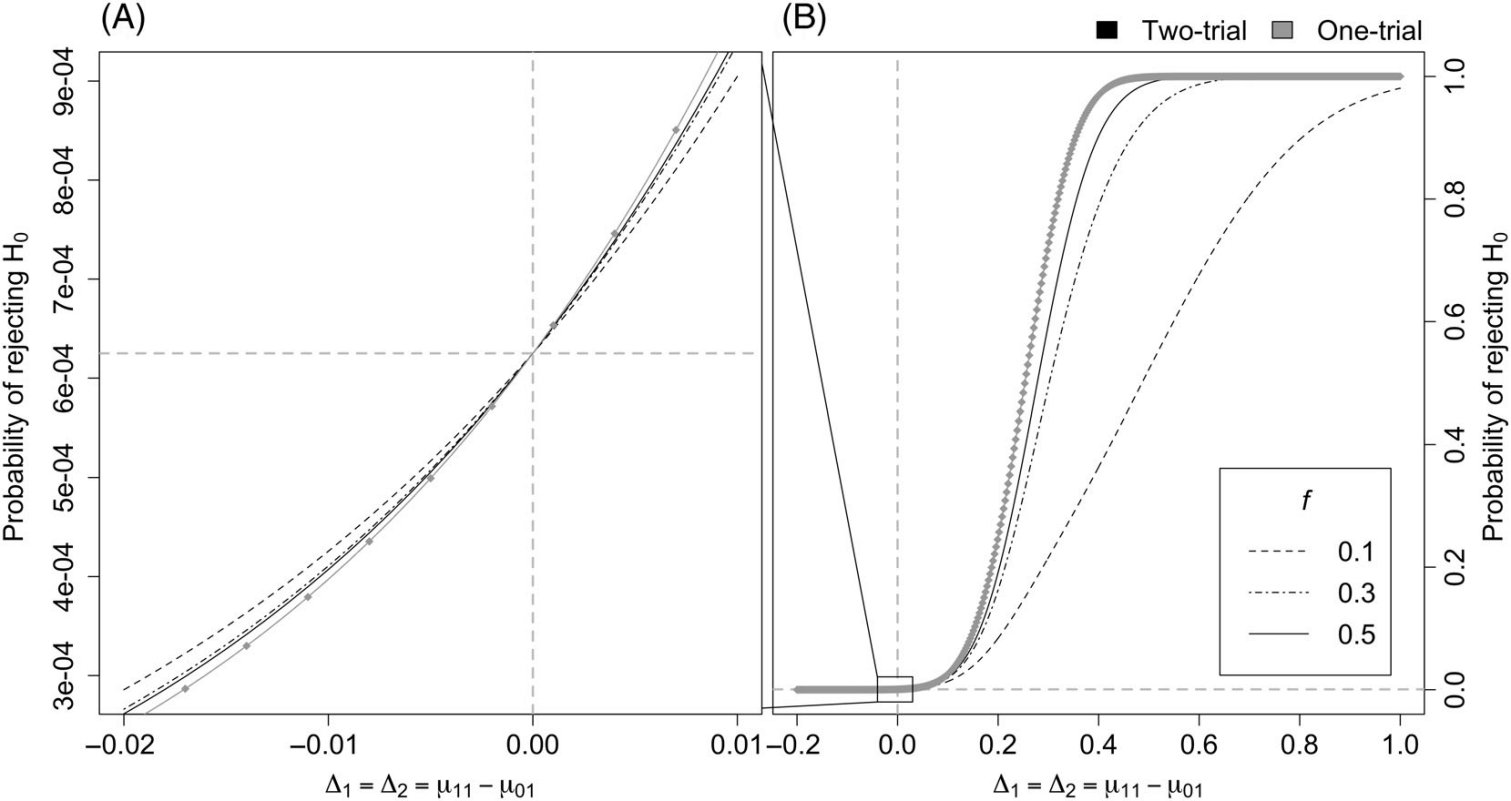
Probability of rejecting *H*_0_ under the “identical populations” scenario (Δ_1_ = Δ_2_) with type I error on the left (A) and power on the right (B). Note that in the two‐trial case, the curve for *f* is the same as the one for 1 − *f*

Under scenario (1), the two individual trials are run in identical populations. Figure 2 represents the probability of rejecting *H*_0_ for the two‐trial (black) and one‐trial (grey with diamonds) rules. The treatment differences Δ_1_ = Δ_2_ are shown on the x‐axis, the probability of rejecting *H*_0_ on the y‐axis is the type I error rate if Δ_1_ ≤ 0 (Figure 2a) and the power if Δ_1_ > 0 (Figure 2b). The different line types indicate various values of *f*. Note that in the two‐trial case, the curve for *f* is the same as the one for 1 − *f*.

The presence of a single grey curve with diamonds in each of the Figures 2a,b confirms that the results for the one‐trial rule are not influenced by *f*. Figure 2a shows that the type I error of the one‐trial rule is never greater than any of the two‐trial curves (black lines) when the effect is negative. Moreover, the type I error of the two‐trial approach gets larger as *f* moves away from 0.5 (solid black line). In all cases, the type I error is below (*α*∕2)^2^ = 0.000625, which is shown by the dashed horizontal line in Figure 2a.

Looking at the power curves in Figure 2b, the one‐trial rule is more powerful than the two‐trial rule regardless of the *f* value. In the two‐trial case, power is higher as the size of the two trials becomes closer to each other (*f* close to 0.5). These results also follows from the fact that the one‐trial approach, under the scenario of identical populations, being a likelihood ratio test, is the uniformly most powerful test in this case. It means that for Δ_1_ > 0, the one‐trial rule provides higher power than the two‐trial rule. For Δ_1_ < 0, one could consider a test with the null value less than zero and the alternative value equal to zero, then the type I error rate must be lower for the one‐trial rule than the two‐trial rule. Our results in Figure 2 show how large the loss in power and the increase in type I error rate are when using the two‐trial rule in this scenario.

Therefore, if it is believed that the two trials are conducted in identical populations, sharing the same population mean, it seems more reasonable to choose the one‐trial approach instead of having two separate trials, regardless of their sizes.

#### 3.2.2 Two different populations

Next, we consider the case when the two trials are conducted in different populations (Δ_1_ ≠ Δ_2_). Since the size of the two populations would influence our results, we consider cases with equal and unequal sample sizes, separately.

##### Equal sizes

**FIGURE 3:**
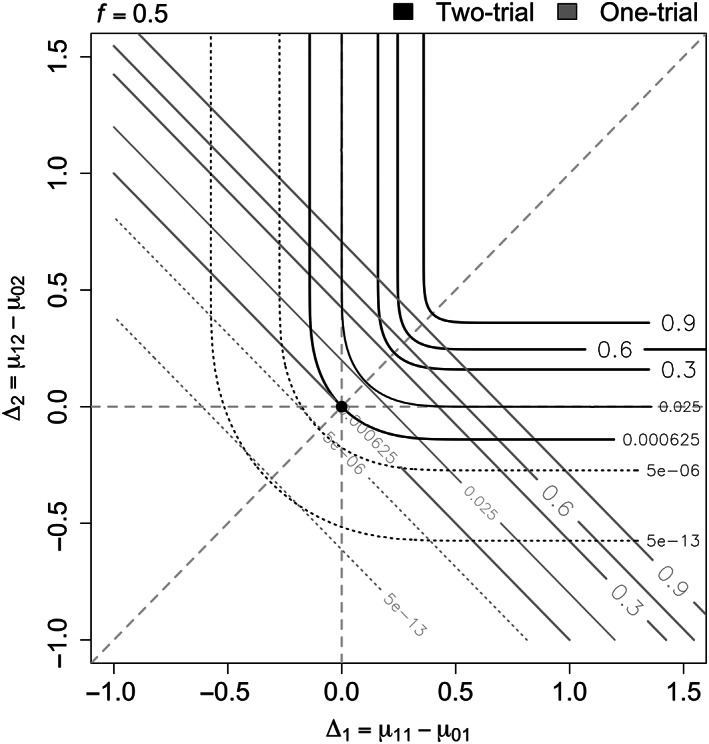
Probability of rejecting *H*_0_ when *f* = 0.5. The two‐trial paradigm is indicated with black lines and the one‐trial paradigm with grey lines

A contour plot displaying the probability of rejecting *H*_0_ in the two paradigms when the two trials are conducted in different populations with the same size is shown in Figure 3. Note that the curves corresponding to *f* = 0.5 in Figure 2 provide the same results as those along the diagonal (dashed grey line) in Figure 3, where Δ_1_ and Δ_2_ are equal.

In particular, it is important to notice that the two strategies have the same overall type I error equal to (*α*∕2)^2^ = 0.000625 only when Δ_1_ = Δ_2_ = 0, as expected. This scenario corresponds to a single point at the boundary of the overall null hypothesis space. As we move away from this point, the two paradigms provide different levels of type I error protection and power.

**FIGURE 4:**
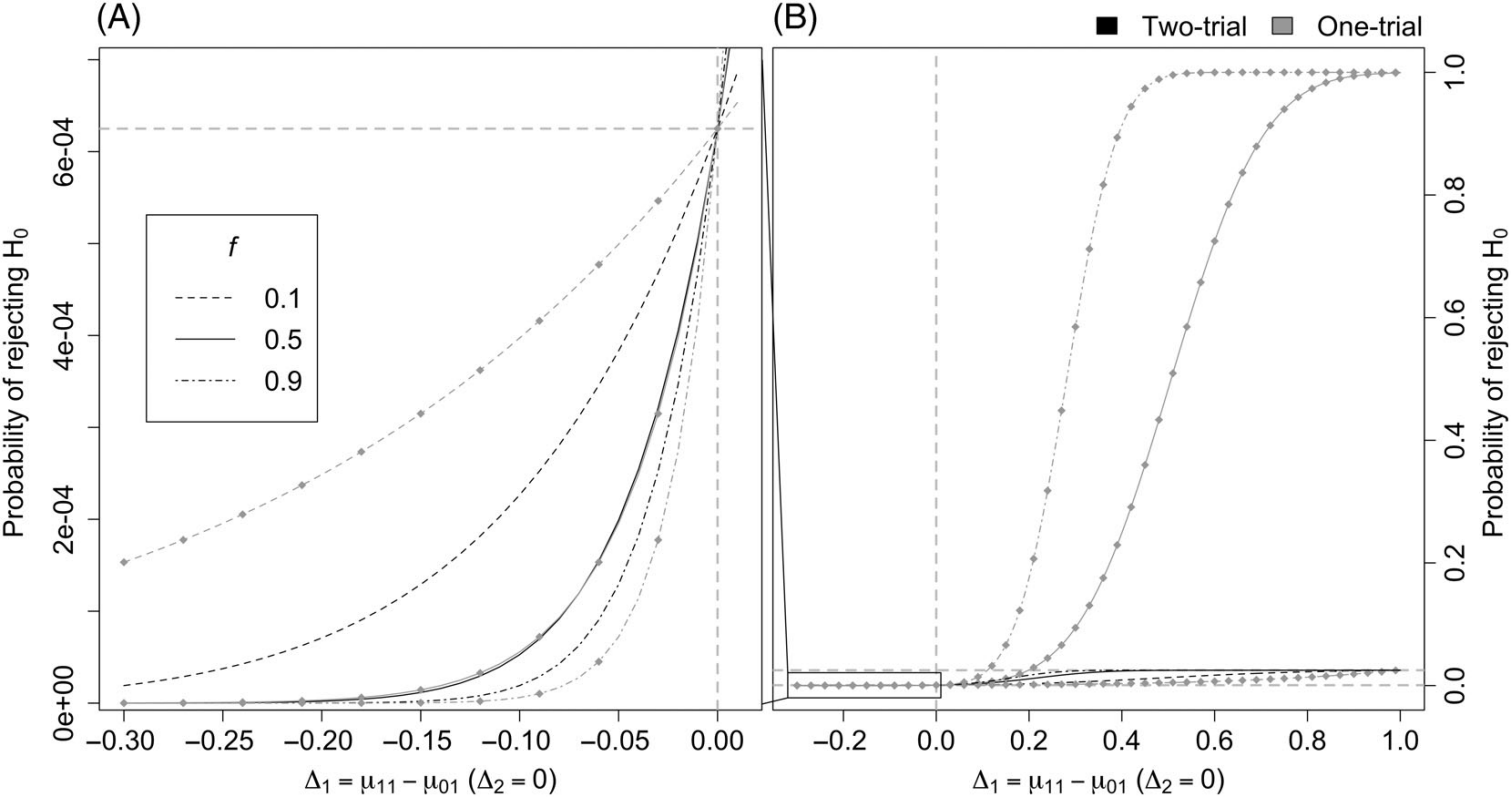
Probability of rejecting *H*_0_ when Δ_1_ ≠ 0 and Δ_2_ = 0 with negative treatment differences (Δ_1_ ≤ 0) on the left (A) and positive treatment differences (Δ_1_ > 0) on the right (B)

Focusing first on the null region with Δ_1_ ≤ 0 or Δ_2_ ≤ 0, we can identify regions where the one‐trial rule has lower type I error rate compared to the two‐trial rule or vice versa (a more detailed plot is provided in Figure S1). As seen in Figure 2, the one‐trial rule has smaller type I error as we move away from (0, 0) along the diagonal. In addition, it is clear from the plot that it has larger type I error as either Δ_1_ or Δ_2_ becomes large with the other ≤ 0. Moving along the vertical or horizontal dashed lines, when one of the treatment differences is equal to zero (Δ_1_ = 0 or Δ_2_ = 0), the two‐trial rule for different populations leads to a maximal type I error of *α*∕2 = 0.025, when the nonzero treatment difference becomes large. The type I error rate moves toward (*α*∕2)^2^ = 0.000625 as the two populations become more similar. Whereas the one‐trial rule, illustrated by the oblique grey lines, can lead to a maximal type I error close to 1 when one of the treatment differences is zero and the other nonzero one becomes large and the type I error decreases to (*α*∕2)^2^ = 0.000625 as the nonzero treatment difference moves toward zero. For example, if we assume that Δ_2_ = *μ*_12_ − *μ*_02_ = 0, the type I error becomes

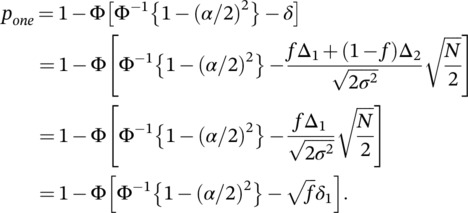

which can take any values between 0 and 1, as Δ_1_ ranges between large negative and large positive values. More detailed plot can be found in Figure 4, where the equal‐sized case is given by the line with *f* = 0.5.

Considering next the alternative region with Δ_1_ > 0 and Δ_2_ > 0 (right upper quadrant in Figure 3). We observe that in all cases the one‐trial rule has higher power than the two‐trial one.

In summary, when two trials are conducted in two different populations with equal size, although the one‐trial rule provides higher power, it also inflates the type I error rate with respect to the union of both individual null hypotheses when one of the treatment differences is zero and the other one becomes large. For this reason, two trials might be better to avoid the high inflation of the type I error rate.

##### Unequal sizes

Now, we move to unequal sample sizes and start with the type I error rates when the treatment effect in one population is zero and the other is nonzero. Figure 4 shows the probability of rejecting *H*_0_ when the treatment difference in population 2 is zero (Δ_2_ = 0) for different values of the true difference in population 1 (Δ_1_). The different line types correspond to the various values of *f* and the line color to the two paradigms (two‐trial in black and one trial in grey with diamonds).

Starting with negative treatment differences for population 1 (Δ_1_ ≤ 0) on the *x*‐axis in Figure 4a, we can observe that the type I error rate is higher for the one‐trial compared to the two‐trial rule when the first trial, in which the effect is negative, is smaller than the second (dashed lines). This can be due to the negative effect of the first trial being not sufficiently captured in the overall data in the one‐trial case, while in two separate trials, there is a lower chance of rejecting the null hypothesis in the smaller trial. When we have a large first trial with *f* = 0.9 (dot‐dashed lines), the two‐trial rule will have higher type I error rate than the one‐trial approach. Note that in all of these cases the type I error rate is less than 0.025^2^ = 0.000625, as indicated by the horizontal dashed grey line.

Moving to positive treatment differences in population 1 (Δ_1_ > 0), shown in Figure 4b, the type I error rates for the two‐trial rule move toward *α*∕2 = 0.025 as Δ_1_ gets larger for all values of *f* (a more detailed plot is provided in Figure S2). Whereas, the one‐trial results increase up to 1 as the nonzero treatment effect becomes larger. For example, when *f* = 0.9 (dot‐dashed grey line with diamonds), the weighting of the smaller trial in the one‐trial approach makes its effect less relevant compared to the larger trial. The positive effect in the first (larger) trial would dominate as the data from the two trials are combined together. Therefore, the one‐trial rule will provide a higher probability of rejecting *H*_0_ compared with the two‐trial case.

**FIGURE 5:**
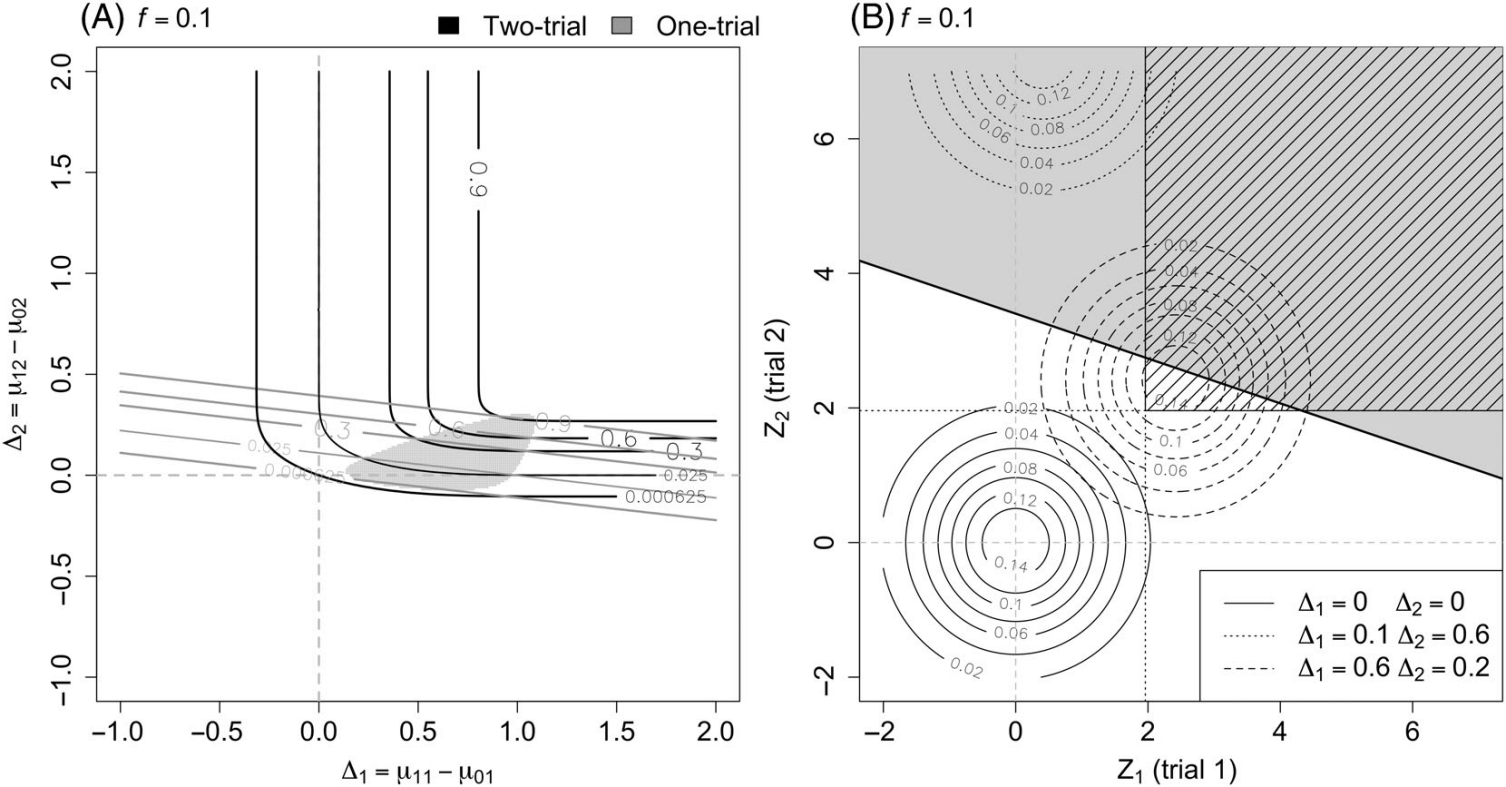
(A) Power contours and (B) distribution of the test statistics with rejection regions when *f* = 0.1 under the scenario of different populations

Figure 5 shows the power contours of the two rules (a) and the distribution of the test statistics (b) for *f* = 0.1, indicating a smaller first trial and larger second trial. In Figure 5a, the region highlighted in grey illustrates pairs of true treatment differences (Δ_1_, Δ_2_) for which the two‐trial rule has higher power than the one‐trial rule. We can observe that a smaller trial with a large effect in combination with a larger trial with smaller effect, would make the two‐trial method more powerful. This happens because in the one‐trial setting the data are considered together and the big effect in the small trial does not heavily contribute to the overall data. Its test statistics are based on the weighted averaged effect which takes into account the sample size of the two trials. When the two trials are kept separate, there is a higher probability of rejecting the null hypothesis with the smaller one and even if there is not a high probability of rejecting with the larger trial, we still have a reasonable chance of rejecting both of them and therefore the two‐trial rule is more powerful.

In Figure 5b, the circles illustrate contours of the density of the bivariate distribution of the test statistics for different pairs of treatment differences. The rejection region for the two‐trial rule is striped and the one for the one‐trial rule is the grey area above the black line. A scenario with a larger effect in a small trial and a smaller effect on the large one (Δ_1_ = 0.6, Δ_2_ = 0.2) is illustrated by the dashed circles. We can see that the gain in power comes from the rejection region of the two‐trial rule that is not in common with the one‐trial rule (striped triangular white area). While, if we have a bigger effect in the second larger trial and smaller effect in the first trial (e.g., when Δ_1_ = 0.1, Δ_2_ = 0.6 as represented by the dotted circles), the one‐trial rule would provide more power.

#### 3.2.3 Heterogeneity between studies

As mentioned in the sections above, the degree of heterogeneity between trials can be measured with the between‐trial variance *τ* ^2^ in a random‐effects model. In addition to the results presented so far, it would be interesting to see how the operating characteristics vary with respect to *τ* ^2^. This can be viewed as a generalization of the two scenarios mentioned in Section 2: *τ* ^2^ = 0 would corresponds to scenario (1) with identical populations and *τ* ^2^ > 0 to scenario (2), when the two populations differ.

**FIGURE 6:**
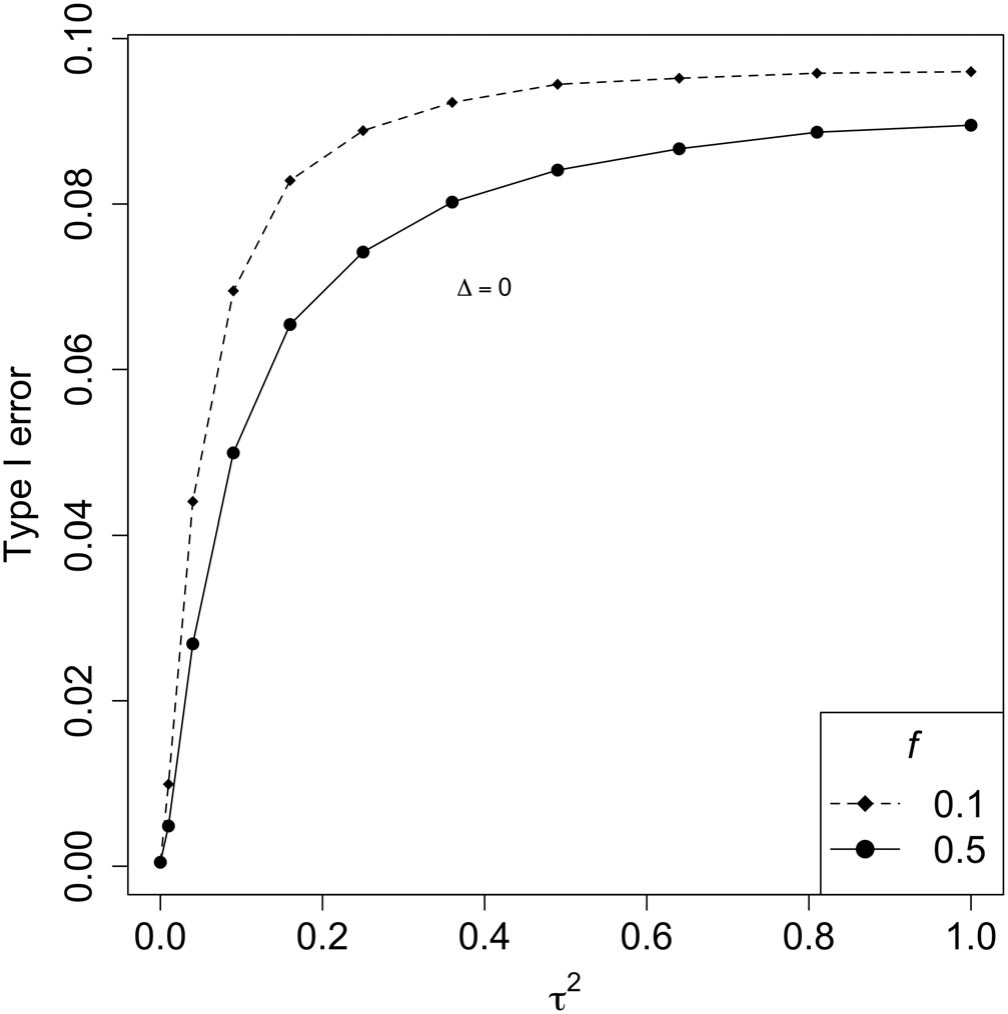
Type I error for different *τ* ^2^ values using the random‐effects meta‐analysis. Different line types indicate the results for various *f* values

**FIGURE 7:**
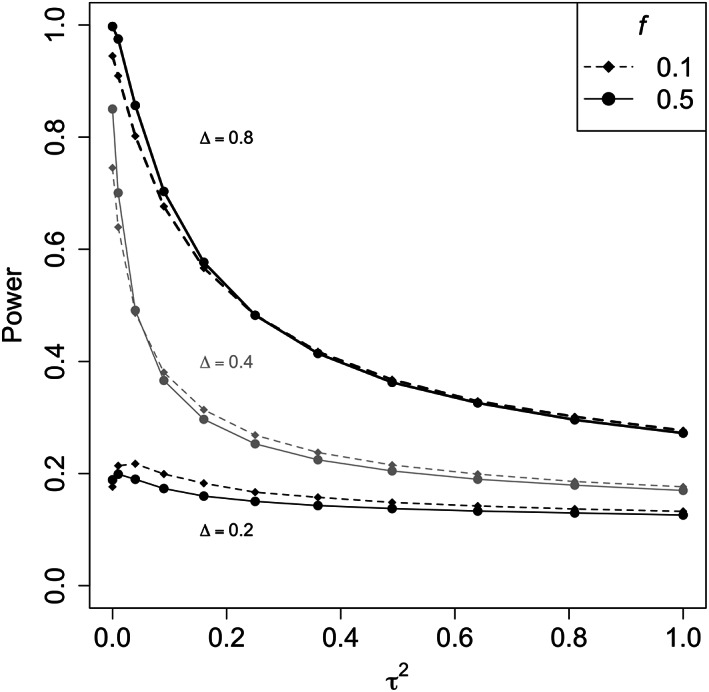
Power for different *τ* ^2^ values using the random‐effects meta‐analysis. Different line types indicate the results for various *f* values

Figure 6 illustrates the type I error rate when assuming a common effect Δ = 0 for different values of *f*. The type I error rate increases as the heterogeneity increases due to a higher chance that one of the treatment effects is positive, with this effect larger when *f* is further from 0.5. Figure 7 shows the power, we can see that the influence of the between‐trial variability is lower for smaller common effects. With higher effects, the power decreases more significantly as *τ* ^2^ increases. The results presented here used the DL estimator for *τ* ^2^, one might also want to compare the results for different estimators of *τ* ^2^ or methods that take into account the uncertainty due to its estimation.^16^, ^17^ However, the estimation remains challenging in the presence of only two trials.

If we assume that *τ* ^2^ is known and fixed, we could compare the results of the random‐effects meta‐analysis with the one‐trial and two‐trial paradigms when the two trials have equal size and one of the treatment effects is zero (see Figure S3). As expected, the curve for the random‐effects model with *τ* ^2^ = 0, which corresponds to the fixed‐effect model, coincides with the results of the one‐trial approach. For negative treatment differences, when Δ_1_ ≤ 0, the type I error rate is higher for larger between‐trial variability and is greater than the one‐trial and two‐trial approaches even with small *τ* ^2^ as 0.1^2^, but is always below the level of (*α*∕2)^2^. With positive treatment differences Δ_1_ > 0, the type I error rate is lower for larger between‐trial heterogeneity. For $\tau^{2}=0.5^{2}$ and 1, the type I error rates are even lower than the two‐trial approach for most of the positive treatment difference Δ_1_, while for *τ* ^2^ = 0.1^2^ the result sits in between the one‐trial and two‐trial curves.

## 4 DISCUSSION

The results above show that the one‐trial and two‐trial paradigms are not directly equivalent. They are testing different hypotheses, resulting in different rejection regions, decision rules, and error rates.

In terms of type I error, the results show that the two rules have the same level of type I error protection only when we are at one specific point in the null hypothesis region (Δ_1_ = Δ_2_ = 0). Under the scenario of identical populations, the one‐trial approach has lower type I error rate compared to the two‐trial approach with neither ever greater than (*α*∕2)^2^ = 0.000625 regardless of the relative sizes of the two trials. Under the scenario of different populations, type I error rates for both approaches reach a maximal value when one treatment difference is zero and the other one becomes large. The type I error rate for the two‐trial rule reaches a maximal value of *α*∕2 = 0.025, while the type I error rate for the one‐trial rule inflates up to 1 with respect to the union of both individual null hypotheses. Although the one‐trial rule would control the type I error rate with respect to the null hypothesis for the average treatment effect, this might not always be of interest. The type I error rate moves toward (*α*∕2)^2^ = 0.000625 as the two populations become more similar, tending to zero as the nonzero treatment effect becomes more negative in both approaches.

In terms of power, Maca et al.^12^ and Shun et al.^13^ have pointed out the gain in power with a larger single trial compared to two independent trials with the same overall alpha level. They showed that the sample size for one trial at *α* level of 0.025^2^ is smaller than the sum of two equally sized trials at *α*∕2 = 0.025, assuming the same overall project power and treatment effect. They have also considered the power with different effect sizes. This is confirmed by our results in the case of identical populations as well as different populations with equal sizes. However, when the sizes of two different populations are not the same, we can identify situations where the two‐trial rule would provide higher power.

Hence, it is important to understand what error the regulators are trying to protect against when using the two‐trial rule. If the aim is to protect against making a claim in a homogeneous population when the effect might be negative, then a single trial at one‐sided level (*α*∕2)^2^ would give better protection. Whereas, if the aim is to protect against making a claim in a heterogeneous population, the two trial paradigm would be more appropriate, though in this case, the two trial populations have to be specified. Although the one‐trial rule is more powerful in some cases, it does not protect against the type I error when one treatment difference is at the boundary of the null space, and the other one is under the alternative.

As introduced at the beginning of the article, according to the FDA guidance, the rationale behind the two‐trial rule is to obtain “independent substantiation of experimental results.” The second study can be obtained via a precise replication of the trial or from studies with a different design investigating different populations, endpoints, or dosage forms. In addition, the FDA claims that trials with differences in design and conduct might offer more informative and persuasive results than two identically designed and conducted trials. At the same time, it supports the idea that there is not a meaningful difference between the strength of evidence provided by a single large multicenter trial and that provided by two smaller trials.^7^ From a statistical point of view, the FDA has not formally stated the problem that they are addressing and what statistical error they are trying to protect against by running two trials (either identically or differently designed and conducted) or running a single large trial.

Another important problem associated with the two‐trial rule is not only the population on which the two trials are conducted (defined by in‐ and exclusion criteria and recruitment preferences) but also other factors that could lead to heterogeneity, for example, trial conduct, sites, countries. As per FDA's suggestion, it seems that running two trials with differences in design and conduct would provide more persuasive evidence. This leads us to think that sometimes we are interested in a broader population from which the two individual trial populations are drawn. Hence, it is important to understand the main aim of running the pivotal trial(s) and what information we would like to gain from them.

An important question is whether we would like to generalize the results obtained from the two trials beyond them or not. If we are restricting ourselves to the trials and interested in a homogeneous population, it seems sensible to conduct the two trials in the same study population or even conduct one single trial. If we run two trials in different study populations and are interested in only one of them, then the effect in the other population would become irrelevant. Whereas, if we are interested in a more general population, within which the sub‐populations have something in common but at the same time present some degree of heterogeneity other than what is expected to occur by chance, in this case, conducting two identical trials in the same population might not give a more persuasive result than two separate trials in different populations. Conducting two completely identical trials at the same time and in similar centers could lead to systematic biases due to study design or center effect, while conducting a large multinational trial could provide a more persuasive result and reduce the influence of trial conduct. At the same time, we need to take into account the requirements of different regulatory agencies, who might be interested in a particular population, instead of a global one (for example, the Japanese PMDA^18^).

It is clear that the one‐trial rule might not be suitable in cases where the interest lies in testing the drug in different settings, therefore more than one trial might be necessary. This might challenge the traditional two‐trial rule: should the required number of pivotal trials be always fixed to two or should it depend on other factors? Could we have alternatives to the two‐trial standard, which can satisfy the evidence required by the regulatory agencies to prove a new drug's efficacy? The Aducanumab example introduced earlier presents a scenario where the approval has been based on the success of one of the two pivotal trials. This can be thought of as a procedure requiring one significant trial out of the two pivotal trials. It can be easily shown that this procedure has a higher overall type I error rate than the standard two‐trial rule or requiring a significant result based on a single pivotal trial. The FDA has not specified the limit of how many trials, out of which we need at least two significant ones, therefore a sponsor could also consider having two significant trials out of three or more trials. Again, the statistical properties of this approach would be different.

If it was considered desirable to run two trials, questions might arise on what would be the optimal strategy in the design of two trials in terms of their size and timing. Based on the results above, in order to maximize the probability of getting significant results in both trials, we could allocate patients based on the effects. For a fixed total number of patients, if we believe that there was a bigger and smaller effect, then it might be sensible to use fewer patients where it is believed that there is a bigger effect and more patients where there is a smaller one. However, to gain a more in‐depth understanding on how to design a pair of studies in two different populations, further investigation is needed, especially by considering whether the trials are run in parallel or in series.

In this article, we have assumed that our trials are run in parallel because this would save time and ensure that they are conducted before the drug's patent expires. Whereas, if we run the trials sequentially, there might be more interesting questions that are worthy of further research, as this could save money and reduce the number of patients involved. Indeed, if one of the two studies is ready or conducted earlier, the results of the first one may be used to influence the design of the second. For example, as suggested by the Associate Editor, we might be interested in the optimal design of a second study for confirmation following a successful result from the first study; as well as the pros and cons of running a single trial at (*α*∕2)^2^ compared with running two trials in series where the first one is successful and the second one turns out to be unsuccessful.

The regulatory substantial evidence requirement of 2 one‐sided *p*‐values <0.025 in two independent studies has its shortcomings. For example, consider the following two scenarios: (1) two *p*‐values are 0.03 and 0.001 (2) two *p*‐values are 0.024 and 0.023. Under the two‐trial rule, only the second scenario would lead to claiming success. However, this does not always reflect the evidence as *p*‐values are also influenced by the sample size. As mentioned previously, under certain circumstances, the one‐trial approach seen as the combination of two trials presents the same test statistics as that of the inverse normal combination test, of which rejection regions are influenced by *f*. With this method, a negative effect in a small trial might be outweighed by a positive effect in a larger trial. To this extent, researchers have also suggested other methods for combining or pooling *p*‐values,^11^, ^13^ such as Fisher's *p*‐value combination method, though again this can present a problem as they could give a significant overall result even when there is a negative trial.

If the only reason behind conducting a second pivotal trial is the need to confirm that the results from the first trial are reproducible, having two trials in different populations might not fulfill this goal. In this regard, Shao et al.^19^ introduced the concept of reproducibility probability as a tool for regulatory agencies in determining the necessity of conducting a second trial or when one trial is sufficient. Recently, Held^20^ proposed a new method for the assessment of replication studies, introducing the concept of the sceptical *p*‐value to measure replication success. Therefore, this work could form a new framework that goes beyond the two‐trial paradigm, questioning the extent to which an independent replication study can confirm the outcome from the first trial. The traditional significance becomes a necessary but not sufficient requirement to define replication success. This new metric would be able to integrate the significance of both trials by taking into account the respective effect sizes. At the same time, a new approach using the harmonic mean *χ* ^2^‐test is proposed for combining one‐sided *p*‐values, which could represent an alternative to the two‐trial paradigm and provide evidence to support drug approval.^21^

## CONFLICT OF INTEREST

The authors declare no potential conflict of interests.

## Supporting information


**Appendix S1** Supplementary Information

## Data Availability

Data sharing is not applicable to this article as no new data were created or analyzed in this study. The R code for reproducing the results in this paper is available at https://github.com/jrzhan07/TwoTrialParadigm.
