# Supplementary material for: Should the two‐trial paradigm still be the gold standard in drug assessment?
Source: Pharm Stat. 2022 Aug 26;22(1):96–111. doi: 10.1002/pst.2262 (PMC10087480; doi:10.1002/pst.2262)
Supplement: Supplementary file 1 — Appendix S1 Supplementary Information [file PST-22-96-s001.pdf]

SUPPORTING INFORMATION

Should the two-trial paradigm still be the gold standard in drug assessment?

Stella Jinran Zhan, Cornelia Ursula Kunz and Nigel Stallard

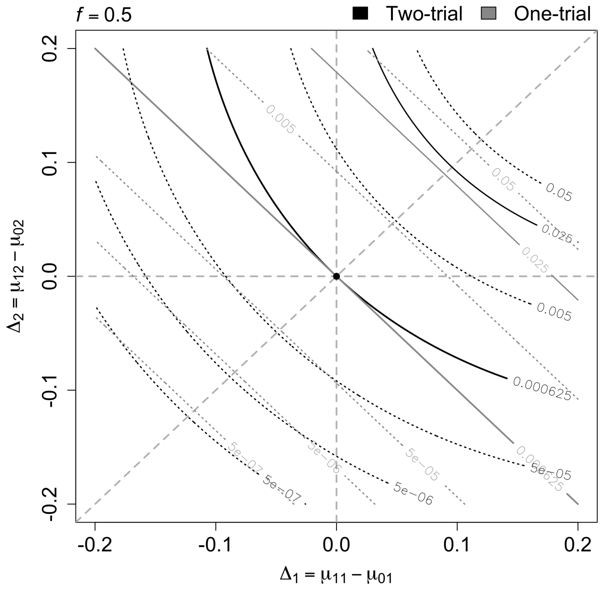

FIGURE S1 Probability of rejecting H_0 when $f = 0.5$, focussing on negative treatment differences. The two-trial paradigm is indicated with black lines and the one-trial paradigm with grey lines.

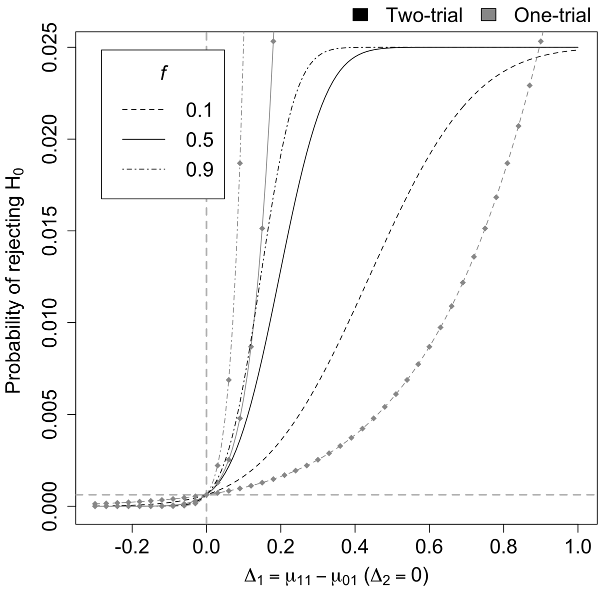

FIGURE S2 Probability of rejecting H_0 when $\Delta_1 > 0$ and $\Delta_2 = 0$, focussing on the results for the two-trial rule.

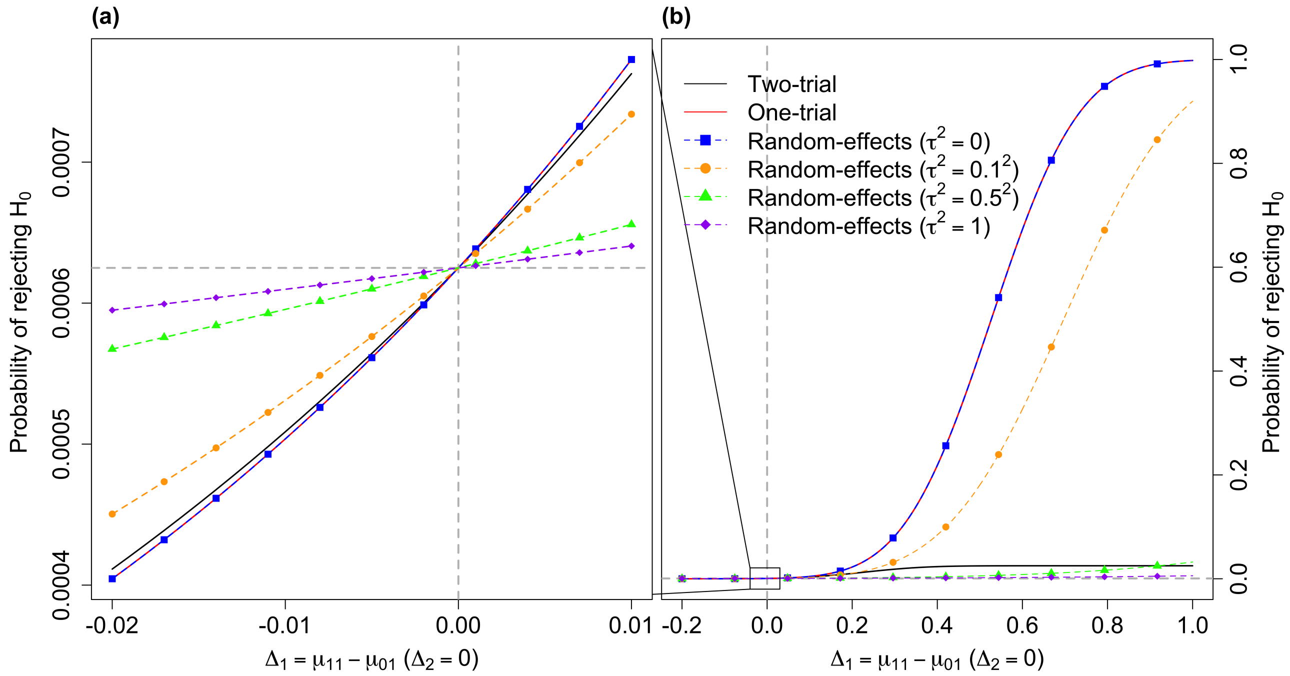

FIGURE S3 Probability of rejecting H_0 when $\Delta_1 \neq 0$ and $\Delta_2 = 0$ with negative treatment differences ($\Delta_1 \leq 0$) on the left (a) and positive treatment differences ($\Delta_1 > 0$) on the right (b). Results for the one-trial, two-trial and random-effects meta-analysis approaches with different between-trial heterogeneity values are included.
